# Solubility Enhancement, Formulation Development, and Antibacterial Activity of Xanthan-Gum-Stabilized Colloidal Gold Nanogel of Hesperidin against *Proteus vulgaris*

**DOI:** 10.3390/gels8100655

**Published:** 2022-10-14

**Authors:** Aftab Alam, Talha Jawaid, Saud M. Alsanad, Mehnaz Kamal, Pinki Rawat, Vinita Singh, Pravej Alam, Prawez Alam

**Affiliations:** 1 Department of Pharmacognosy, College of Pharmacy, Prince Sattam bin Abdulaziz University, P.O. Box 173, Al-Kharj 11942, Saudi Arabia; 2 Department of Pharmacology, College of Medicine, Imam Mohammad Ibn Saud Islamic University (IMSIU), Riyadh 13317, Saudi Arabia; 3 Department of Pharmaceutical Chemistry, College of Pharmacy, Prince Sattam bin Abdulaziz University, Al-Kharj 11942, Saudi Arabia; 4 Maharana Pratap College of Pharmacy, Kanpur 209217, Uttar Pradesh, India; 5 IES Institute of Pharmacy, IES University Campus, Kalkheda, Ratibad Main Road, Bhopal 462044, Madhya Pradesh, India; 6 Department of Biology, College of Science and Humanities, Prince Sattam bin Abdulaziz University, Al-Kharj 11942, Saudi Arabia

**Keywords:** hesperidin, *Proteus vulgaris*, antibacterial, xanthan gum, gold nanoparticles

## Abstract

The objective of the study was to develop a transdermal nanoformulation of hesperidin (HSP) against *Proteus vulgaris* (*P. vulgaris*). Based on the low water solubility of HSP, we prepared HSP-enabled AuNPs stabilized with xanthan gum (XA), referred to as HSP@XA@AuNPs. The HSP@XA@AuNP formulation was evaluated for particle size (43.16 nm), PDI (0.565), zeta potential (−31.9 mV), and entrapment efficiency (56.7%). The HSP@XA@AuNPs gel was developed by incorporating selected formulation grades into a 1% Carbopol gel base and characterized by physical evaluation and rheological studies. The color of the HSP@XA@AuNP gel was light pink, and the texture was very smooth and non-greasy. The gel was shown to be odorless. A field emission scanning electron microscope (FESEM) was used to investigate the shape of HSP@XA@AuNPs further. The drug release was 73.08% for the HSP@XA@AuNPs and 86.26% for the HSP@XA@AuNPs gel in 500 min. The prepared gel showed antimicrobial activity against *P. vulgaris* with an MIC of 1.78 μg/mL. In conclusion, the HSP@XA@AuNPs gel could be an advanced modality for treating *P. vulgaris*.

## 1. Introduction

Proteus is a Gram-negative bacterium that thrives in the soil, water, and digestive tract of mammals and can swarm or flounder on solid surfaces. As a Gram-negative, motile, and non-spore-forming bacterium, *Proteus vulgaris* (*P. vulgaris*) belongs to the genus Proteus of the family Enterobacteriaceae [1,2]. Several Proteus bacteria have colonized and infected human hosts, but the most common are associated with causing human disease. *P. vulgaris* is closely associated with clinical practice and is the most important causative agent of urinary tract infections (UTIs), which pose a serious threat of community and hospital infections [3]. Previously, *P. vulgaris* was considered a biogroup that causes wounds, burns, bloodstream infections, and respiratory infections [4,5,6].

The consumption of herbs is being performed more consciously because the natural compounds are effective against various microorganisms. The industry involved in the treatment of antimicrobial diseases has welcomed the increasing use of drugs based on plants and plant components [7,8,9]. In line with this philosophy, hesperidin (HSP) is widely used in herbal therapies. HSP is a flavanone glycoside formed from hesperetin aglycone and the disaccharide rutinose. Citrus fruits contain the plant pigment HSP. In addition to citrus, HRP has been found in several other plant families, including Fabaceae, Betulaceae, Laminaseae, and Papilionaceae [10]. It has been described as having a variety of biological activities, including antioxidant, anti-inflammatory, antiallergic, antihypertensive, antimicrobial, anticarcinogenic, and vasodilator properties [11,12,13]. Long-term epidemiological studies have shown that people who regularly eat a flavonoid-rich diet have a lower risk of cancer and other chronic diseases. This has increased interest in using these compounds as dietary supplements for treating diseases [14].

However, the water solubility of HSP is very low, resulting in limited transmembrane permeability and insufficient bioavailability. Regardless, its water solubility is negligible, a critical problem in formulation development. It is poorly absorbed in the small intestine, which significantly reduces the bioavailability of the drug [15]. Therefore, applications in functional foods, beverages, dietary supplements, pharmaceuticals, etc., are severely limited [16]. These specific solubility limitations also affect various diseases’ bioavailability and treatment benefits. To achieve a better pharmacological effect, HSP’s solubility in solutions needs to be increased. It has been claimed that different carrier systems improve the solubility of HSP drugs. Nanotechnology makes it possible to provide traditional natural phytocomponents with low solubility. By combining traditional natural products with nanometer-sized entities, we can overcome many problems—such as stability, solubility, and toxicity—often associated with traditional compounds and also provide a platform for targeted delivery to disease sites. For example, to improve solubility, stability, and bioactive potential, Ali et al. prepared a formulation based on hesperidin–PLGA–poloxamer 407 [17]. This will allow researchers to solve the existing limitations of the formulation by applying various nanotechnology/nanomaterial-based approaches [18,19]. Various nanocarriers such as lipid-based nanocarriers [20], solid lipids [21], liposomes [22], niosomes [23], and metal nanoparticles [24] have been reported to solve the problems related to low water solubility and bioavailability of therapeutic compounds.

Among these various nanomaterials mentioned above, metal nanoparticles are used in biomedical applications [25]. In particular, gold nanoparticles (AuNPs) have been the focus of interest due to their apparent advantages. The advantages of multi-surface functionality reinforce their wide application in nanotechnology. Similarly, a slight change in the dimensions, appearance, surface framework, and interparticle spacing leads to well-controlled changes in the physical and chemical properties of the above nanostructured materials, especially for drug delivery [26]. AuNPs are inherently biocompatible and exhibit higher drug loading and improved pharmacological response [27]. In the synthesis of AuNPs, countless scientists have found that the physical and chemical character of nanoparticles changes [28,29]. Stabilizing AuNPs with biodegradable polymers and gums is an innovative field of study for synthesizing more suitable nanomaterials. Recently, several researchers have synthesized various natural polymers and gums to produce hybrid AuNPs with higher stability and better disease targets [30,31].

In this context, we selected xanthan gum (XA) as a stabilizing agent for AuNPs loaded with HSP. Inorganic NPs of iron [32], silver [33], silicon [33], palladium [34] and gold [35,36] were recently prepared and stabilized with XA.

In addition, many researchers have experimented with Carbopol-based inorganic metal/metal oxide nanoparticles and hybrid delivery systems with organic additives in various disease states. Carbopols are polymers of acrylic acid linked to polyalkyl ethers and dimethyl glycols. Due to their hydrophilic properties, the cross-linked structure of Carbopols is a potential candidate for use as a gel-like formulation for current use in transdermal drug delivery. For example, Jana et al. used Carbopol gel with chitosan–egg albumin nanoparticles for transdermal delivery of aceclofenac [37]. Similarly, Martínez-Higuera et al. formulated a Carbopol-based hydrogel as a matrix for the dispersion of *Mimosa tenuifora* extract and Ag nanoparticles to improve the healing of burn wounds and prevent infections [38]. Transdermal application of drugs has many advantages over oral and intravenous methods. These include the lack of first-pass metabolism and widespread toxicity. In addition, these systems have been shown to improve patient compliance due to ease of use and the ability to discontinue treatment if needed [39].

To prove this hypothesis, a simple approach to synthesize Au nanoparticles loaded with HSP and stabilized with XA (HSP@XA@AuNPs) was adopted in this study. In the present study, we attempted to develop a transdermal Carbopol gel containing HSP@XA@AuNPs. After preparation and characterization, we tested the antibacterial activity of the HSP@XA@AuNPs gels against *P. vulgaris*.

## 2. Results and Discussion

### 2.1. Solubility Study

We carried out the solubility study of HSP in water, buffer solution, and XA-stabilized AuNPs, as given in Table 1. From the table, it is clear that the solubility of HSP is reported to be low in water (4.09 ± 0.20 µg/mL), pH 1.2 (2.44 ± 0.12 µg/mL), pH 5.0 (3.42 ± 0.17 µg/mL), and pH 6.8 (6.19 ± 0.30 µg/mL). On the other hand, we find that the solubility is increased by 15-fold by the addition of XA-stabilized AuNPs compared to water. This indicates the enhancement of solubility by adding XA-stabilized AuNPs. The data of the solubility study are given in Table 1. In this study, hesperidin’s aqueous solubility was improved in the presence of XA-stabilized AuNPs. Here, we observed a sharp increase in the HSP’s solubility mainly due to the influence of the particle size of the incorporated XA-stabilized AuNPs. The reduction in particle size generally leads to a faster dissolution rate as the surface area increases [40]. An increase in surface area with a reduction in NP size promotes dissolution due to an increase in surface area that can participate in the dissolution process.

### 2.2. Drug Capping, Particle Size, and Zeta Potential Measurements

**Figure 1:**
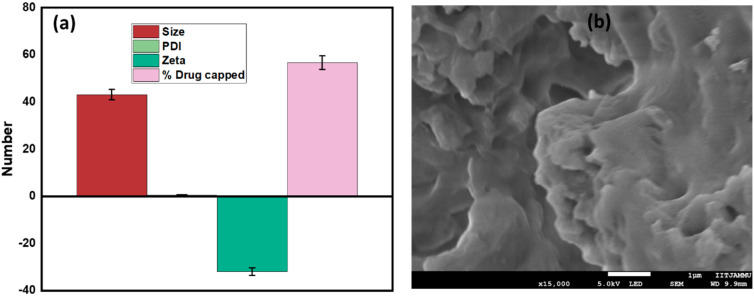
Characterization of HSP@XA@AuNPs: (**a**) particle size, PDI, and zeta potential; (**b**) morphology of HSP@XA@AuNPs gel determined by FESEM technique.

The percentage of drug capped was found to be 56.7 ± 2.835%. The drug loading calculated was 34.27 ± 0.23%. The high drug load in HSP@XA@AuNPs suggests that XA@AuNPs are more effective than HSP. The particle size of XA@AuNPs was reported to be 18.3 ± 30 nm, with PDI of 0.502 ± 0.076 and −29.2 mV. In this study, the particles were smaller than those reported previously [41], i.e., 43.16 ± 2.158 nm, with a PDI of 0.565 ± 0.028 (Figure 1a). The zeta potential of the surface charge was −31.9 ± 1.595 mV. The corresponding method shows that the charged nanoparticles are stable in solution [42]. The surface charge of the particles and the binding properties of the drug NP are the main factors that determine the absorption rate of the drug in the NP and the loading efficiency. The ZP values can also indicate whether the loaded material is encapsulated in the NP center or on the surface [43]. These results are consistent with the previously published work of Sulaiman et al. [19].

### 2.3. Organoleptic, Morphological, and Rheological Study of HSP@XA@AuNPs Gel

The organoleptic observation was made by examining changes in the gel preparation’s shape, color, and odor. The color of the HSP@XA@AuNPs gel was light pink, and the texture was very smooth and non-greasy. The gel was found to be odorless. A field emission scanning electron microscope (FESEM) was used to investigate the shape of the HSP@XA@AuNPs gel further. FESEM analysis of the HSP@XA@AuNPs gel showed an irregular shape (Figure 1b). From the FESEM images, it can be seen that the particles have an irregular shape or no particular morphology. During the quality control test, the prepared gel formulations were examined for their physical appearance, pH, viscosity, spreadability, and extrudability (Table 2). Acidity (pH) is an essential parameter in gel preparations because the gel is a topical preparation applied to the skin. Therefore, gel preparations must have a pH equivalent to human skin (4.5–6.5) to avoid skin irritation or redness. This test shows that the pH of the gel preparation is 6.34 ± 1.56, which is still within the pH range of human skin [44]. The spreadability of gel preparations is defined as the ability of the gel to spread on the skin surface. The spreadability of the HSP@XA@AuNPs gel was determined to be 4.19 ± 1.78 cm. Viscosity is a measure of the thickness of a liquid; a gel preparation refers to liquids with high viscosity of 2000–4000 cps [45]. The viscosity was determined to be 3031 cps. Viscosity is inversely proportional to spreadability; the higher the viscosity, the lower the spreadability.

### 2.4. In Vitro Cell Viability Assay

**Figure 2:**
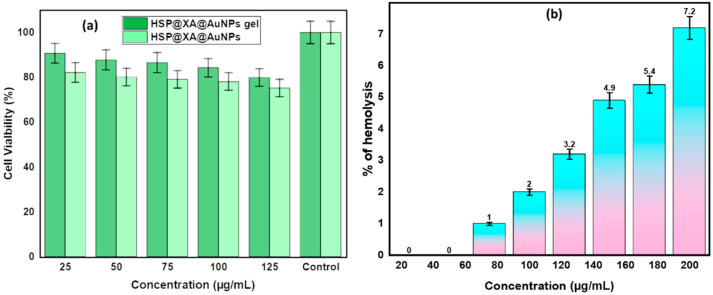
(**a**) In vitro cell viability assay of HSP@XA@AuNPs gel and HSP@XA@AuNPs in HaCaT cells; (**b**) in vitro hemolysis assay of HSP@XA@AuNPs gel.

Biosafety is an essential criterion for determining nontoxic dosage formulations for biomedical applications. Cytotoxicity testing is widely regarded as a rapid and efficient method for evaluating the biocompatibility of biomaterials. A cytotoxicity study based on the MTT assay was used to evaluate and quantify the cytotoxicity of HSP@XA@AuNPs and the HSP@XA@AuNPs gel formulation. Cell viability results using the MTT assay explain how cells respond to a toxic agent. In the present study, the synthesized HSP@XA@AuNPs and HSP@XA@AuNPs gel showed cell viability of more than 75% (Table 3 and Table 4) and Figure 2a. This shows that the material is not toxic to mammalian cells. These results show that the HSP@XA@AuNPs and the HSP@XA@AuNPs gel can be considered a nontoxic and safe drug delivery system.

### 2.5. In Vitro Hemolysis Assay

For preclinical safety testing of the HSP@XA@AuNPs gel, we performed an in vitro hemolysis study, as shown in Figure 2b. The result of the hemolysis study shows that the HSP@XA@AuNPs gel is biocompatible up to a concentration of 160 µg/mL, which is less than 5%. After this concentration, the hemolysis percentage increases with increasing concentration from 180 µg/mL, indicating an incompatibility problem. This is based on the rules of the ASTM E2524-08 standard, according to which a hemolysis value higher than 5% indicates that the tested nanoparticles damage erythrocytes [40]. The same standard states that AuNPs and HSP–AuNPs are hemocompatible, which means that can be used in the body, but only in low concentrations.

### 2.6. Drug Release Studies

**Figure 3:**
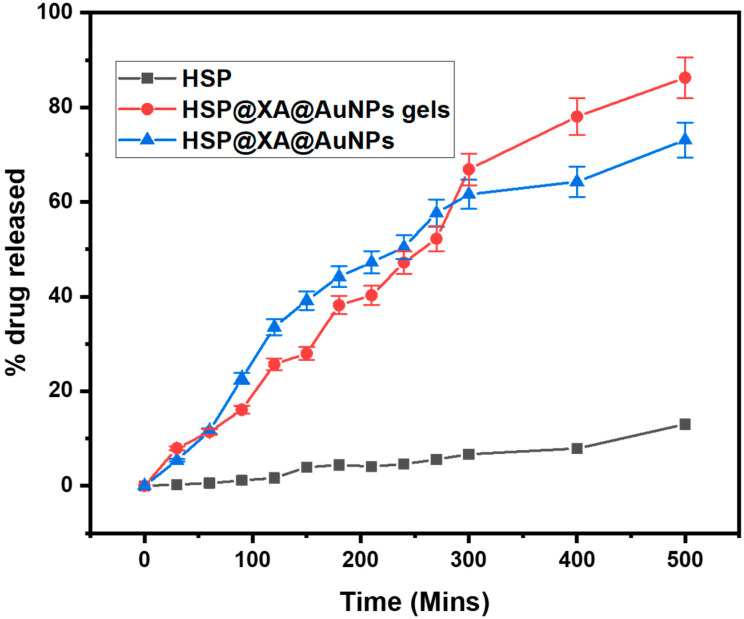
Comparative in vitro drug release study of HSP, HSP@XA@AuNPs gel, and HSP@XA@AuNPs in 500 min.

The relationships between the cumulative release rate and contact time for HSP@XA@AuNPs gel and HSP@XA@AuNPs are shown in Figure 3. It can be seen that the HSP@XA@AuNPs gel has a better release performance compared to HSP@XA@AuNPs. It was observed that initially, the drug release was higher in HSP@XA@AuNPs (50.43 ± 2.52) and HSP@XA@AuNPs gel (47.19 ± 2.35) within 240 min. After 240 min, the drug release gradually increased in the case of HSP@XA@AuNPs gel. The drug release was 73.08 ± 3.65 and 86.26 ± 4.31 for HSP@XA@AuNPs and HSP@XA@AuNPs gel in 500 min, respectively. The drug release pattern showed a biphasic release pattern, an initial explosive release followed by a sustained release of HSP in the case of HSP@XA@AuNPs gel. The inconsistent release of HSP at the beginning of dissolution indicates a delay in the transition to the gel. The continued sustained release of the drug indicates slower drug diffusion from the gel matrix.

### 2.7. Minimum Inhibitory Concentration

*P. vulgaris* (MTCC 7299) was selected for the MIC study. Based on the obtained results, the HSP@XA@AuNPs gel loaded with HSP showed higher MIC values for the selected bacterial species than the nanoparticles. This indicates that the XA@AuNPs solution was less effective as an antibacterial agent than the HSP@XA@AuNPs gel, as shown in Table 5. For instance, metal NPs are pivotal bases of ions toxic to bacterial cells and constantly discharge ions after interrelating through the bacterial cell wall [46]. Notably, HSP@XA@AuNPs gel showed more excellent antibacterial activity than XA@AuNPs and HSP. These results showed that incorporating HSP@XA@AuNPs into gel had synergistic antibacterial properties. Results suggested that HSP@XA@AuNPs gel was the most potent combination against *P. vulgaris*. Consequently, targeting the *P. vulgaris* by considering XA@AuNPs gel as a drug carrier could be a good alternative.

## 3. Conclusions

In summary, XA-stabilized AuNPs loaded with HSP were prepared and evaluated. The formulation has a suitable shape and size and the highest encapsulation efficiency. Finally, the HSP@XA@AuNPs gel formulation was mixed into a Carbopol gel and thoroughly evaluated for homogeneity, spreadability, pH, viscosity, in vitro release, and minimum inhibitory concentration. The results indicate that the HSP@XA@AuNPs gel is a promising antibacterial drug carrier for the treatment of *Proteus vulgaris*.

## 4. Materials and Methods

HSP, HAuCl_4_·3H_2_O, XA, and di-sodium hydrogen phosphate dihydrate were purchased from Sigma-Aldrich. Dimethyl sulfoxide (DMSO), MTT, trypsin, ethanol, EDTA, and phosphate-buffered saline (PBS) were purchased from Sigma Chemicals. All water solutions were prepared from high-purity water filtered with Milli-Q Plus equipment (Millipore Co.). The solvents and analytical chemicals used were highly pure and were not further processed. Cells are purchased from National Centre for Cell Science (NCCS, Pune, India).

### 4.1. Preparation of XA Solution

The XA stock solution was prepared by mixing 500 mg of XA with 50 mL water and 50 mL ethanol. It was shaken overnight at room temperature. The solution was centrifuged to remove insoluble material, and the supernatant was lyophilized. The dry freeze-dried powder was dissolved in water to obtain the desired XA concentration whenever necessary [36].

### 4.2. Preparation of Gold Chloride Solution

To obtain gold chloride solutions, 3.96 mg of gold chloride was dissolved in 0.5 mL Milli-Q water and 0.5 mL ethanol. It was kept in a magnetic stirrer for 25 min to dissolve it.

### 4.3. Synthesis of Gold Nanoparticles (AuNPs)

Using previous methods, the stabilized colloidal AuNPs were synthesized by sliding modifications [30]. In the synthesis, 100 µL of HAuCl_4_ solution (10 mM) was added to 5 mL of XA solution (0.5%). Then, the temperature was maintained at 80 ± 5 °C for 30 min and stirring was continued for 1 h. One hour after starting the experiment, the color was found to change from white to ruby red. This was used to demonstrate the synthesis of AuNPs.

### 4.4. Capping of HSP into XA-Stabilized AuNPs

To cap the XA@AuNPs with HSP, the required mixture was prepared by adding 10.0 mg of HSP to 10 mL of XA@AuNP solution and stirring the mixture at a speed of 1000 rpm for 60 min. After the solution was prepared, it was placed in an incubator at room temperature for 24 h to load the HSP onto the XA@AuNPs. The solution was centrifuged at 15,000 rpm for half an hour. HSP@XA@AuNPs were separated from the supernatant solution, and the collected pellet was redistributed in Milli-Q water before use. The HSP concentration of the supernatant was estimated from the λmax value at 286 nm and measured using a UV-Vis spectrophotometer (Shimadzu). The entrapment efficiency and loading capacity of hesperidin were calculated according to the following Formula (1):

(1) $$\%\;\mathrm{Drug}\;\mathrm{capping}=\frac{\left(\mathrm{Total}\;\mathrm{amount}\;\mathrm{of}\;\mathrm{HSP}\;\mathrm{added}-\mathrm{Amount}\;\mathrm{of}\;\mathrm{free}\;\mathrm{HSP}\;\mathrm{in}\;\mathrm{supernatent}\right)}{\mathrm{Total}\;\mathrm{amount}\;\mathrm{of}\;\mathrm{HSP}\;\mathrm{added}}\times 100$$

The final nanosolutions were freeze-dried and stored in airtight containers at 4 °C. The formulation of the experiment was performed in triplicates.

The drug loading (DL) was determined by measuring the amount of drug released when the known HSP@XA@AuNPs were completely dissolved in the PBS. The concentration of freeze-dried HSP@XA@AuNPs was determined following redispersion in deionized water. The drug percentage loading was determined by UV spectrophotometry using Equation (2):

(2) $$\mathrm{Drug}\;\mathrm{loading}==\frac{\mathrm{Weight}\;\mathrm{of}\;\mathrm{HSP}\;\mathrm{in}\;\mathrm{XA}@\mathrm{AuNPs}}{\mathrm{Weight}\;\mathrm{of}\;\mathrm{XA}@\mathrm{AuNPs}}\times 100$$

### 4.5. Solubility Study

The solubility of HSP was determined by adopting the standard shake flask technique according to the method described by Majumdar and colleagues [47]. The screening of the solution was performed by evaluating the solubility of HSP (10% (*w*/*v*) with respect to pH solution. To perform this experiment, 10% *w*/*v* of HSP was added to a glass vial containing 5 mL of the chosen solvent and tightly sealed. To ensure homogeneous mixing, the samples were shaken continuously for 24 h in a water bath at room temperature and 50 rpm. After 24 h, samples were centrifuged at 4500 rpm and HSP was assayed in the supernatant. Solubility was determined in water and in buffers with pH values ranging from 1.2 to 6.8. In addition, the effect of XA-stabilized AuNPs on the solubility of hesperidin was examined to determine by how much the solubility was increased.

### 4.6. Particle Size and Size Distribution Measurements

Particle size, polydispersity index (PDI), and zeta potential were measured at 25 °C using a Malvern Zetasizer Nano ZS (Malvern Instruments, UK). After diluting the dispersion of HSP@XA@AuNPs to a sufficient amount with deionized water, the diameter was determined. All samples were analyzed in triplicate [31].

### 4.7. Gel Formulations of HSP@XA@AuNPs

First, we prepared the dispersion of Carbopol 940. For this, 100 mL of distilled water was placed in a beaker, and 1 g of Carbopol 940 was added to it. This mixture of water–Carbopol 940 was stirred continuously to form a dispersion. After the dispersion was complete, the triethanolamine was added dropwise to produce a clear gel formulation. Then, 100 mg of HSP@XA@AuNPs gel was observed for several parameters [41]. After this, the prepared HSP@XA@AuNPs were incorporated into Carbopol 940 dispersion. It was stirred for 15 min, and further characterization was carried out.

### 4.8. Rheological Study of HSP@XA@AuNPs Gel

Viscosity: HSP@XA@AuNPs gels were stored at room temperature before each measurement. Viscosity was measured using a Brookfield viscosity monitor. The gel formulation was cut at relatively low speeds (0-3-6 rpm), which did not damage the gel structure. The viscosity was read directly from the viscometer display.

pH: The pH meter uses standard buffer solutions (pH 4.0–7.0). After 0.5 g of gel was weighed and dissolved in 50 mL of distilled water, the pH was measured.

Spreadability: The 0.5 g sample of gel was weighed and pressed between two horizontal plates (20 × 20 cm). Then a 500 g weight was placed on the upper plate and left for about 5 min. The diameter of the dispersion circle was measured in centimeters. The results obtained were the average of three determinations.

### 4.9. Morphology

A field emission scanning electron microscope (FESEM; JEOL JSM-6490LV) was used to study the morphology or shape of the HSP@XA@AuNPs gels. For this purpose, the sample was placed on a copper stub with double-sided adhesive tape. Then the sample was sputtered with gold at 20 mA for 120 s and analyzed in the FESEM (JEOL JSM-6700F, Japan) at an excitation voltage of 5 kV [48].

### 4.10. In Vitro Cell Viability Assay

The MTT assay for cell viability was performed using HSP@XA@AuNPs and HSP@XA@AuNPs gels in a human epidermal keratinocyte line, i.e., HaCaT cells, by following the technique reported in [49,50]. Cells were grown in Dulbecco’s modified Eagle medium (DMEM) supplemented with 10% (*v*/*v*) heat-activated fetal bovine serum. Cells were maintained at 37 °C in a humidified incubator with 5% CO_2_. For subculture, cells were harvested by trypsinization when 80% confluence was reached and divided (1:4). The growth medium was changed every 3 days. Percent cell viability was calculated according to Equation (3).

(3) $$\%\mathrm{Cell}\;\mathrm{viability}=\frac{\left(A570\;\mathrm{nm}\;\mathrm{treated}\;\mathrm{cells}\right)}{\left(A570\;\mathrm{nm}\;\mathrm{untreated}\;\mathrm{cells}\right)}\times 100$$

### 4.11. Hemolysis Test

The biocompatibility of HSP@XA@AuNPs was verified by blood hemolysis tests. The procedure of the previously described technique was slightly modified [17]. Briefly, 100 µL of whole blood was mixed with 700 µL of PBS. A 96-well round bottom plate containing 100 μL of suspension was treated with various concentrations of nanocarriers ranging from 20 to 200 μg/mL. The plate was then gently stirred and incubated at 37 °C for 1 h. Finally, all mixtures were centrifuged at 700 rpm for 5 min and the absorbance rate was read (541 nm, UV-Vis spectrophotometer). The percentage of hemolysis was calculated using Equation (4). Here, At is the absorbance of the treated supernatant, Ac is the absorbance of the negative control, and Ax is the absorbance of the positive control.

(4) $$\%\mathrm{Hemolysis}=\frac{\left(\mathrm{At}-\mathrm{Ac}\right)}{\left(\mathrm{Ax}-\mathrm{Ac}\right)}\times 100$$

### 4.12. Drug Release Studies

The in vitro drug release study was performed according to the USP method. To perform the HSP release study, we used USP I by holding it at a speed of 50 rpm in 500 mL of pH 6.0 at 37 ± 0.5 °C in 500 min. The selected 2 mL samples of HSP, HSP@XA@AuNPs gel, and HSP@XA@AuNPs were added to the dialysis tubing (dialysis bag, pore size 14,000 Da) after one end was tightly sealed. Then the other end was sealed and the bag was placed in the basket immediately. Samples were collected at a specific interval, and HSP concentration was determined spectrophotometrically at 286 nm.

### 4.13. Minimum Inhibitory Concentration

*Proteus vulgaris* (Gram-negative bacteria) (MTCC 7299) was selected, and MIC was determined in sterile microtiter plates using the method given by Yousef et al. [51]. Briefly, stock solutions of XA@AuNPs gel and HSP@XA@AuNPs gel were prepared in water to ensure complete solubilization at a concentration of 1 mg/mL. A total of 100 µL of nutrient broth and Sabouraud dextrose broth was added to wells 1 to 10. XA@AuNPs gel and HSP@XA@AuNPs gel samples (100 µL) were added to the first well. The solution was serially diluted from well 1 to well 10, while 100 µL from well 10 was discarded. Then 100 µL of the bacterial suspension was added to all dilution wells from well 1 to well 10. The overnight bacterial suspension (100 µL) was added to well 11, and 100 µL of sterile broth was added to serve as a positive control or growth control, while 200 µL of sterile nutrient broth and Sabouraud dextrose broth in well 12 served as a negative control or sterility control. The plate was incubated at 37 °C for 24 h. After incubation, absorbance in each well was measured using an ELISA reader (Erba) at a wavelength of 640 nm. The procedure described above was performed for each microbial strain. The concentration of the sample and standard that inhibited 50% of bacterial growth was determined for all microorganisms. All tests were performed in triplicate to minimize error.

## Figures and Tables

**Table 1:** Solubility study of HSP.

Solvent	Hesperidin Solubility (µg/mL)
Water	4.09 ± 0.20
pH 1.2	2.44 ± 0.12
pH 5.0	3.42 ± 0.17
pH 6.8	6.19 ± 0.30
XA-stabilized AuNPs	62.23 ± 3.1

**Table 2:** Rheological characteristics of HSP@XA@AuNPs gels and HSP@XA@AuNPs.

Formulation	Clarity	Homogeneity	pH	Viscosity (cps)	Spreadability (cm)
Carbopol gel	Clear	Homogeneous	5.5 ± 1.24	3000	3.76 ± 2.13
HSP@XA@AuNPs gel	Clear	Homogeneous	6.34 ± 1.56	3031	4.19 ± 1.78
HSP@XA@AuNPs	Transparent	Homogeneous	7.0 ± 0.64	100	NA

**Table 3:** Observation of cell viability study of HSP@XA@AuNPs gel.

Concentration (µg)	Absorbance at 570 nm	% Viability
25	0.564	90.757
50	0.546	87.822
75	0.538	86.572
100	0.524	84.262
125	0.478	79.909
Untreated	0.622	100
Blank	0	0

**Table 4:** Observation of cell viability study of HSP@XA@AuNPs.

Concentration (µg)	Absorbance at 570 nm	% Viability
25	0.511	82.17
50	0.498	80.12
75	0.492	79.12
100	0.485	78.12
125	0.467	75.234
Untreated	0.622	100
Blank	0	0

**Table 5:** Comparative MIC determination of the different compounds.

Compound	MIC (μg/mL)
XA@AuNPs	3.12
HSP@XA@AuNPs	1.95
HSP@XA@AuNPs gel	1.78
HSP powder	9.2
Ofloxacin (standard)	0.19

## Data Availability

The data presented in this study are available on request from the corresponding author.
